# Adiponectin serum levels and *ADIPOQ* (rs2241766) polymorphism in alopecia areata Egyptian patients^⋆^

**DOI:** 10.1016/j.abd.2023.05.003

**Published:** 2023-11-18

**Authors:** Azza Gaber Antar Farag, Eman Abd-Elfatah Badr, Banan Mohamed Gamal Abd-Elaty, Nada Farag Elnaidany, Mai Medhat Mohamed Ghanem

**Affiliations:** a Dermatology, Andrology and STDs Department, Faculty of Medicine, Menoufia University, Shebin El-Kom, Menoufia, Egypt; b Medical Biochemistry and Molecular Biology Department, Faculty of Medicine, Menoufia University, Shebin El-Kom, Menoufia, Egypt; c Dermatology Department, Sers El-Lian General Hospital, Ministry of Health, Sers El-Lian, Menoufia, Egypt; d Clinical Pharmacy department, Faculty of Pharmacy, Modern Sciences and Arts University, 6TH October, Egypt

**Keywords:** Adiponectin, Alopecia areata, Genetics

## Abstract

**Background:**

Alopecia Areata (AA) is an acquired autoimmune form of non-scarring hair loss. Adiponectin and its gene polymorphism were related to many autoimmune disorders.

**Objective:**

Assessment of adiponectin serum levels and adiponectin gene (*ADIPOQ*) (rs2241766) Single Nucleoid Polymorphism (SNP) in AA patients and correlating the results with the disease severity in those patients.

**Methods:**

This study included 75 AA patients and 75 age and gender-matched healthy subjects (controls). The severity of Alopecia Tool (SALT) score assessment to evaluate AA severity was done. Adiponectin serum levels by ELISA and *ADIPOQ* (rs2241766) SNP using PCR were performed.

**Results:**

Adiponectin serum levels were significantly lower in AA patients than controls (p = 0.001). *ADIPOQ* (rs2241766) *TG* genotype and *G* allele were significantly predominant in AA patients increasing its risk by 5 and 4 folds (OR = 5.17, p = 0.001), (OR = 3.82, p = 0.001) respectively. Serum adiponectin levels were negatively correlated with SALT score (*r* = -0.435, p = 0.001) and associated with alopecia totalis (p = 0.016). *ADIPOQ* (rs2241766) *TG* genotype was significantly associated with low serum adiponectin levels and higher SALT score (p = 0.001).

**Study limitations:**

The small sample size.

**Conclusions:**

*ADIPOQ* (rs2241766) gene polymorphism (*TG* genotype and *G* allele) may modulate AA risk and contribute to the development of AA in Egyptian populations. Decreased circulating adiponectin levels may have a dynamic role in AA etiopathogenesis. Adiponectin serum concentration can be considered a severity marker of hair loss in AA.

## Introduction

Alopecia areata represents a type of hair loss, in which the immune system mistakenly attacks hair follicles.^1^ The estimated prevalence of AA is approximately 1 in 1000 people, with a lifespan risk of about 2%.^2^ Both adults and children are affected by AA, with a similar rate in females and males.^3^

The exact etiology of AA remains elusive. It is considered an autoimmune disorder. The immune system in its entirety is influenced by many genetic and multiple environmental factors.^4^ AA is associated with an increased risk of metabolic comorbidities, suggesting that adipokines may play a role in AA pathogenesis.^5^

Adipose tissue is not an inert tissue. It actively produces adipokines. These adipokines, in addition to regulation of energy expenditure and insulin sensitivity, play an important role as regulators of many physiologic and pathologic processes, comprising inflammation and immunity.^6^

Adiponectin is an adipose tissue circulating adipokine that enhances insulin sensitivity.^7^ It is a protein having 224 amino acids. Adiponectin structure is made of a single-chain of trimmers; a variable N-terminal domain, a C-terminal globular domain, and a collagen domain. This trimer is enclosed by a bell-shaped structure.^8^

Adiponectin exerts many pleiotropic actions; it promotes insulin sensitivity, promotes apoptosis in carcinogenic cells, and has antioxidant and anti-inflammatory effects.^9^

A decreased blood level of adiponectin has been found in psoriasis,^10^, ^11^ acne vulgaris,^12^, ^13^, ^14^ and atopic dermatitis.^15^ However, in AA only two recent studies were interested in investigating adiponectin serum levels in AA patients. Moreover, the two author groups reported controversial results.^16^, ^17^

*ADIPOQ* is located on the long arm of the chromosome 3, locus 3q27.^18^ This gene is 17 kb long and consists of 3 exons and 2 introns.^19^ *ADIPOQ* SNPs are associated with an increased risk of many diseases including atherosclerosis,^20^ rheumatoid arthritis,^21^ acne vulgaris^19^ and atopic dermatitis,^22^ but have not evaluated in AA until now.

The aim of this study was to elucidate the possible role of adiponectin in AA through assessment of *ADIPOQ* (rs2241766) gene polymorphism in relation to its serum level in a sample of Egyptian patients having AA, as well as correlate the evaluated results with clinical aspects of AA including its site, course and disease severity in those patients.

## Methods

The current case-control study was conducted on a total number of 150 subjects. They included 75 patients with AA (36 males and 39 females) and 75 aged and sex-matched apparently healthy volunteers (43 males and 32 females) as a control group. They were collected from the Dermatology Outpatient Clinic, Menoufia University Hospital during the period from March 2022 to February 2023. The laboratory part of the study was done at the Medical Biochemistry and Molecular Biology Department, Faculty of Medicine, Menoufia University.

### Administrative and ethical design

• A written consent was obtained from all participants after being informed about the aim and process of the study as well as applicable objectives.
• The study had been approved by the local ethics committee on research involving human subjects of Menoufia Faculty of Medicine. The ethical approval number is 3/2022 DERMA 19.
• The study procedures were free from any harmful effects on the participants as well as the service provided.
• The principal investigator has kept individual data as private information safely. There was no extra fee to be paid by the participants and the investigator covered all the costs in this regard.

Inclusion criteria were patients with variable degrees of AA severity from both sexes and aged 18 years or more.

Any subject had one or more of the following was excluded from the study: a) Patients with a history of using any treatment that could impact the metabolic status within 3 months prior to the examination e.g., hormonal therapy and systemic treatment such as oral prednisolone therapy. b) Patients with other autoimmune diseases e.g. Hashimoto's thyroiditis and vitiligo. c) Pregnant and lactating women.

### Methods

Each of the selected cases was subjected to history taking and complete general examination. Body Mass Index (BMI) was calculated [BMI = weight (kg)/height (m)^2^].^23^

Diagnosis of AA was done clinically by 2 expert dermatologists. Distribution of the AA lesions was evaluated by determination of its site (scalp, beard, eyebrow, and/or any hairy areas all over the body). Clinical variants of AA (patchy AA, A totalis, A universalis, ophiasis, sisaipho) were recorded. Clinical assessment of the disease severity and extent was done based on SALT score.^24^ In which the scalp was divided into four sections: right (18%), left (18%), back (24%) and top (40%). For each section, the percent of the area of the hair involved was estimated and then transformed into a grade from S0 to S5. SALT score was calculated by multiplying the percentage of hair loss in each of the 4 quadrants of the scalp by the quadrant surface area. Then the 4 values were added together for a total score. The SALT score ranged from 0% to 100%.

After 12 hours fasting, six mL of venous blood were collected from every participant, under complete aseptic condition. Each sample was divided into 2 parts. One (3 mL) for DNA extraction was kept in EDTA tube. The other part (3 mL) was put in a plain tube, left to clot for 30 minutes at room temperature, then underwent centrifugation for 10 minutes at 4000 rotations per minute and the serum obtained was divided into a liquor, stored at −80 °C until the time for Fasting Blood Glucose (FBG), lipid profile and serum adiponectin analysis.

Blood glucose was determined by enzymatic colorimetric test. Glucose is oxidized by glucose oxidase to glucuronic acid and hydrogen peroxide. Then, the formed hydrogen peroxide is detected by a chromogenic oxygen acceptor, phenol aminophenazone in the presence of peroxidase: produces Quinonimine which is a colored compound the intensity of the color is directly proportional to the amount of glucose in the sample. The absorbance of the sample and standard test was measured by a spectrophotometer instrument and the concentration of the glucose in the sample was determined.^25^

Glucose + O_2_ + H_2_O ----- glucose oxidase-- → H_2_O_2_ + Gluconate

2H_2_O_2_ + phenol + 4-Ap ------- peroxidase --- → Quinonimine + 4H_2_O

Quantitative estimation of Total Cholesterol (TC), High-Density Lipoprotein (HDL) and Triglyceride (TG) using colorimetric enzymatic method, using standard enzymatic colorimetric kits (Spinreact diagnostic kit, Spain) and low-density lipoprotein was elaborated by Modified Friedewald et al. equation.^26^

Detection of adiponectin serum level was done by enzyme-linked immunosorbent assay (ELISA). The kit (made in China, Sun Red Company) uses a double-antibody sandwich ELISA.

Assessment of *ADIPOQ* (rs2241766) genetic variants was carried out by real-time PCR. DNA extraction from whole blood by Quick-genomic DNATM MiniPrep kit, Zymo Research. Genotyping of *ADIPOQ (rs2241766)* was completed by allelic discrimination assay utilizing TaqMan probes (Applied Biosystems, USA). An overall mixture of 25 μL was conducted by applying 5 μL of sample DNA to a mixture of 12.5 μL of genotyping master mix, 1.25 μL of SNP assay and 6.25 μL of nuclease-free water. The TaqMan probes were labeled with VIC and FAM ﬂuorescent dyes. The probe sequence for *ADIPOQ (rs2241766)* was TTCTACTGCTATTAGCTCTGCCCGG [T/G] CATGACCAGGAAACCACGACTCAAG; Cycling provisions were completed as: initial 95 °C for 10 minutes as a primary denaturation step followed by 45 cycles of 15 seconds at 95 °C and 60 seconds at 60 °C (cycling), and a final extension step for 60 seconds at 60 °C. The Sequence Detection System implements the fluorescence emitted during the plate read and the fluorescence (Rn) values were plotted depending on the signals from each well. Each well of the 96-well reaction plate is an individual point on the plot. Fluorescence detection and data analysis were carried out by 7500 Real-Time PCR instrument (Applied Biosystems) version 2.0.1.

### Statistical analysis

Data were collected, tabulated, and statistically analyzed using an international business machine corporation personal computer with Statistical Package of Social Science (SPSS) version 22 (SPSS, Inc, Chicago, Illinois, USA). The following statistics were applied: a) Descriptive statistics: in which quantitative data were presented in the form of mean, Standard Deviation (SD), range, and qualitative data were presented in the form of numbers and percentages. b) Analytical statistics: was used to find out the possible association between studied factors and the targeted disease. The used tests of significance included: Chi-Square test (χ2),^27^ Student *t*-test (*t*),^28^ Mann-Whitney test (*U*),^29^ Kruskal Wallis test^30^; p-value of was considered significant if it was ≤ 0.05.

## Results

### Personal and clinical data of the investigated subjects

AA patients were 36 (48%) males and 39 (52%) females with a male: female ratio of 1:1.08. Their age ranged from 22 to 53 years with 32.4 ± 7.14 years as a mean ± SD value. Control subjects were 43 (57.3%) males and 32 (42.7%) females with a male: female ratio of 1.34:1. Their age ranged from 18 to 54 years with 32.7 ± 9.11 years as a mean ± SD value. There were non-statistically significant differences between cases and controls regarding their age (p = 0.956) and sex (p = 0.252). Table 1 demonstrates the clinical data of AA patients (n = 75).

**Table 1:** Personal and clinical data of AA patients (n = 75).

Studied variables	NO	%
AA patients		
(No = 75)		
**Gender**		
Male	36	48
Female	39	52
**Age / years**		
Mean ± SD	32.4 ± 7.14	
Median	31.0	
Range	22.0 – 53.0	
**Onset**		
Sudden	95	78.7
Gradual	16	21.3
**Course of the disease**		
Progressive	44	58.7
Stationary	31	41.3
**Duration / months**		
Mean ± SD	11.3 ± 6.80	
Median	10.0	
Range	2.00 – 36.0	
**Family history**		
Positive	9	12.0
Negative	66	88.0
**Type of alopecia**		
Patchy	71	94.7
Totalis	3	4.00
Ophiasis	1	1.30
**Associations**		
Anxiety	53	70.7
Depression	6	8.00
Atopy	1	1.30
No	15	20.0
**Nail changes**		
Beau line	3	4.00
Nail bitting	4	5.30
Nail ridding	3	4.00
Punctuate leukonychia	3	4.00
Trachyonychia	2	2.70
No	60	80.0
**SALT score**		
Mean ± SD	17.2 ± 31.9	
Median	1.80	
Range	0.60 – 100.0	

No, Number; %, Percentage; SD, Standard Deviation; AA, Alopecia Areata; SALT, Severity of Alopecia Areata Tool.

### BMI, blood pressure and laboratory investigations of the studied subjects

There were non-significant differences between AA patients and controls regarding their BMI (p = 0.131), systolic blood pressure (p = 0.665), diastolic blood pressure (p = 0.304), fasting blood glucose (p = 0.171), triglycerides (p = 0.097) and low-density lipoprotein (p = 0.347). While there was a significant elevation in total cholesterol (p = 0.019) and a significant decrease in high-density lipoprotein (p = 0.001) (Table 2).

**Table 2:** Comparison between AA patients and controls regarding their BMI, blood pressure and laboratory investigations (n = 150).

Studied variables	AA patients (No = 75)	Controls (No = 75)	Mann Whitney test	p-value
**BMI (**kg/m^2^)				
Mean ± SD	24.7 ± 3.96	22.3 ± 2.04		
Median	24.0	22.0	1.51	0.131
Range	18.0 – 35.0	19.0 – 26.0		
**Systolic blood pressure** (mmHg)				
Mean ± SD	118.5 ± 10.0	118.4 ± 9.65		
Median	120.0	120.0	0.432	0.665
Range	90.0 – 135.0	90.0 – 135.0		
**Diastolic blood pressure** (mmHg)				
Mean ± SD	80.9 ± 5.85	79.8 ± 5.60		
Median	80.0	80.0	1.02	0.304
Range	60.0 – 90.0	60.0 – 90.0		
**Fasting Blood Glucose (mg/dL)**				
Mean ± SD	88.3 ± 8.22	91.6 ± 11.7		
Median	89.0	90.0	1.36	0.171
Range	73.0 – 103.0	73.0 – 125.0		
**Triglyceride (mg/dL)**				
Mean ± SD	128.7 ± 13.9	123.4 ± 21.3		
Median	129.0	125.0	1.65	0.097
Range	100.0 – 152.0	89.0 – 170.0		
**Total cholesterol (mg/dL)**				
Mean ± SD	149.8 ± 24.0	140.5 ± 22.4		
Median	150.5	140.0	2.34	0.019*
Range	111.0 – 196.0	110.0 – 200.0		
**HDLc (mg/dL)**				
Mean ± SD	66.2 ± 4.15	73.7 ± 6.69		
Median	66.0	75.0	6.86	0.001*
Range	60.0 – 77.0	60.0 – 88.0		
**LDLc (mg/dL)**				
Mean ± SD	87.8 ± 9.09	85.8 ± 10.6		
Median	90.0	87.0	0.941	0.347
Range	69.0 – 102.0	47.0 – 104.0		

SD, Standard Deviation; U, Mann Whitney test; BMI, Body Mass Index; FBG, Fasting Blood Glucose; TG, Triglyceride; HDL, High Density Lipoprotein; DL, Low Density Lipoprotein; AA, Alopecia Areata.

### Serum adiponectin levels and *ADIPOQ* (rs2241766) of the studied subjects

Serum adiponectin levels weresignificantly lower in AA patients than in controls (9.45 ± 4.12 vs. 13.8 ± 5.62 ng/mL) (p = 0.001) (Table 3).

**Table 3:** Comparison between AA patients and controls regarding serum adiponectin levels and *ADIPOQ* (rs2241766) gene SNP.

Studied variables	AA patients (No = 75)		Controls (No = 75)		Test	p-value	OR (95% CI)
Serum adiponectin levels (ng/mL)					Mann-Whitney		
Mean ± SD	9.45 ± 4.12		13.8 ± 5.62		4.38	**0.001**	**‒**
Median	9.60		12.6				
Range	2.80 – 15.3		6.89 ‒ 29.5				
*ADIPOQ* (rs2241766) genotypes	**No.**	**%**	**No.**	**%**	**χ^2^**		
*TT*	44	58.7	66	88.0			Reference **5.17 (2.24 – 11.9)**
*TG*	31	41.3	9	12.0	16.5	**0.001**	
**Alleles**	**N = 150**	**%**	**N = 150**	**%**	**χ^2^**		
*T*	119	79.3	141	94.0	13.9	**0.001**	Reference **3.82 (1.75 – 8.35)**
*G*	31	20.7	9	6.00			

AA, Alopecia Areata; No., Number; %, Percentage; χ^2^, Chi square test; OR, Odds Ratio; CI, Confidence interval.

Studying *ADIPOQ* (rs2241766) gene polymorphism showed that there was a significant difference between AA patients and controls. *TG* genotype was present in 31 (41.3%) AA patients versus 9 (12.0%) controls. The presence of *TG* genotype significantly increased the risk for AA by about 5 folds (OR = 5.17, p = 0.001). Also, *G*allele significantly increases the risk for AA by about 4 folds (OR = 3.82, p = 0.001) (Table 3).

### Adiponectin serum levels in relation to studied data of AA patients

**Figure 1:**
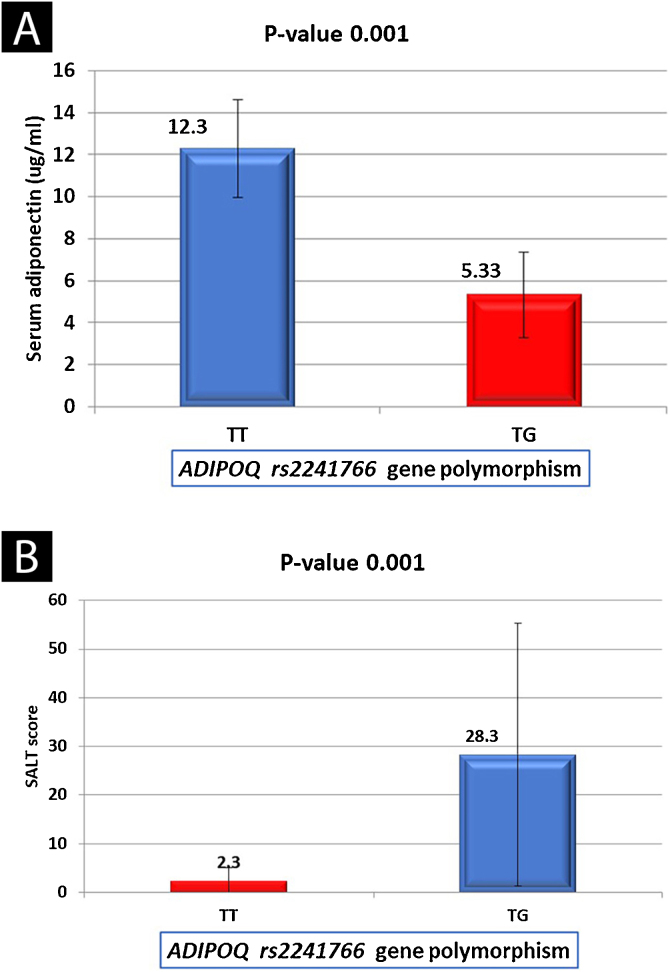
*ADIPOQ* (rs2241766) genotypes in relation to: (A) Serum adiponectin levels among the studied AA patients. (B) SALT score of studied AA patients.

There were non-significant relations between serum adiponectin level and the investigated data of AA patients except for the type of alopecia (significantly lower in alopecia totalis [p = 0.016]) and SALT score [a significant negative correlation (*r* = -0.435, p = 0.001) (Table 4). Serum adiponectin level was significantly lower in AA patients with *TG* genotype than in cases having *TT* genotype (5.33 ± 2.02 vs. 12.3 ± 2.34) (p = 0.001) (Fig. 1A).

**Table 4:** Relation between serum adiponectin level and studied data of AA patients (n = 75).

Studied variables	Serum adiponectin	Test of significance	p-value
	Mean ± SD (ng/mL)		
**Gender**			
Male	9.07 ± 3.82	**U**	0.373
Female	9.81 ± 4.39	0.891	
**Onset**			
Sudden	9.55 ± 4.12	U	0.605
Gradual	9.11 ± 4.22	0.518	
**Course of the disease**			
Progressive	8.93 ± 4.70	**U**	0.287
Stationary	10.2 ± 3.02	1.06	
**Family history**			
Positive	10.4 ± 3.75	U	0.546
Negative	9.32 ± 4.17	0.603	
**Type of alopecia**			
Ophiasis	11.4 ± 0.00		
Patchy	9.69 ± 4.01	K	0.016*
Totalis	3.10 ± 0.26	8.31	
**Associations**			
Anxiety	9.90 ± 3.68		
Depression	6.48 ± 4.98	K	0.088
Atopy	3.40 ± 0.00	6.55	
**Nail changes**			
Beau line	13.8 ± 0.92		
Nail bitting	9.85 ± 6.28	K	0.229
Nail ridding	11.2 ± 2.82	5.62	
Punctuate leukonychia	3.70 ± 0.51		
Trachyonychia	8.25 ± 4.45		
**Age/years**	**r**		**p-value**
Age/years	0.183		0.115
**Disease duration/months**	0.025		0.833
**BMI (**kg/m^2^)	0.110		0.348
**SBP** (mmHg)	-0.080		0.497
**DBP** (mmHg)	0.122		0.340
**Fasting Blood sugar (mg/dL)**	0.171		0.143
**Triglyceride (mg/dL)**	-0.085		0.471
**Total cholesterol (mg/dL)**	0.143		0.220
**HDLc (mg/dL)**	0.048		0.685
**LDLc (mg/dL)**	-0.032		0.787
**SALT score**	-0.435		**0.001**

SD, Standard Deviation; K, Kruskal Wallis test; U, Mann Whitney test; *r, Spearman’s correlation; BMI, Body Mass Index; SBP, Systolic Blood Pressure; DBP, Diastolic Blood Pressure; HDL, High Density Lipoprotein; LDL, Low Density Lipoprotein; SALT, Severity of Alopecia Areata Tool.

### Relationship between *ADIPOQ* (rs2241766) genotypes and studied data of AA patients

There were non-significant relationships between *ADIPOQ* (rs2241766) genotypes and any of the clinical data of studied AA patients except for the SALT score that was significantly higher in *TG* genotype carriers than *TT* carrier cases (p = 0.001) (Fig. 1B).

## Discussion

In the current study, *ADIPOQ* (rs2241766) SNP was strongly related to adiponectin serum concentrations in AA patients, and associated with the disease severity. By this study, we have shownthat *ADIPOQ* (rs2241766) SNP could modulate AA risk and contribute to the development of AA in Egyptian populations that had not previously been identified, and we confirmed that decreased circulating adiponectin levels may have an active role in AA etiopathogenesis. Confirmatory studies are necessary to make sure that the observed associations are true rather than spurious.

Metabolism and the immune system are connected via a network of numerous soluble mediators known as adipokines. These adipokines are regarded as bad and good adipokines. Bad adipokines are pro-inflammatory and produce insulin resistance, vascular dysfunction, and local inflammatory reactions that support cutaneous inflammation. On the other hand, good adipokines have opposed properties.^31^

Here in, we considered adiponectin as a good adipokine. We found a significant decrease in its circulating levels in AA patients than controls, and this low concentration was more observed with more severe disease and in alopecia totalis cases.

Confirming these observations, Stochmal et al.^17^ demonstrated a significant decrease in adiponectin serum levels in AA patients than their matched peers. They also reported that adiponectin serum level was negatively correlated with SALT score and had its lowest concentration in patients with alopecia universalis. The decreased levels of serum adiponectin were also described in patients with Sjogren’s syndrome, psoriatic arthritis, and multiple sclerosis.^32^

Adiponectin, a serum protein, is produced mostly by adipocytes. But, other cells such as the fibroblasts, endothelial cells, macrophages, and leukocytes also may synthesize adiponectin.^33^ Adiponectin consumes an anti-inflammatory influence. It reduces B-cell lymphopoiesis and T-cell responsiveness, as well as TNF-α synthesis. It also stimulates IL-10 production.^32^

AA pathogenesis is not completely assumed. It appears that immune system activation is considered one of the main biological processes connected to the pathogenesis of AA.^34^ In AA the unusual levels of many pro-inflammatory cytokines e.g. IL-6 and TNF-α and anti-inflammatory ones as IL-10 were confirmed.^35^, ^36^ It appears that cytokine production disequilibrium, with a relative excess of pro-inflammatory versus low anti-inflammatory cytokines, could have an active role in AA development.^37^

TNF-α is a normal in vitro inhibitor of hair follicle growth. TNF-α, together with IL-1α and -β, induces matrix cell vacuolation within the bulb of the hair follicle and decreases the matrix size. It also disorganizes the follicular melanocytes and induces abnormal differentiation in the inner root sheath and precortical cells.^38^ It was reported that TNF-α serum^39^ and tissue^38^ levels in patients with AA were significantly higher than controls.

Thus, we hypothesized that in AA, adiponectin could act as an anti-inflammatory molecule, and the current demonstrated low adiponectin serum levels in AA patients may be translated into a repressed anti-inflammatory activity. Impaired adiponectin levels may influence numerous processes within the hair follicle environment leading to local autoimmune response resulting in exacerbated hair loss.

However, Serarslan et al.^16^ did not show significant differences in adiponectin concentration in patients with AA compared to healthy controls. Moreover, we showed higher serum levels of adiponectin in patients with scalp hair loss compared to those with isolated AA in the beard and eyebrow. The authors said that their result was an unexpected finding. They added that, as in RA, where adiponectin serum and synovial levels were increased, adiponectin is suggested to increase the production of inflammatory mediators.^40^ These controversial results need more similar large-scale studies to verify.

In the present work we analyzed, for the first time, *ADIPOQ* (rs2241766) SNP in AA patients, and correlated its genotypes with the AA severity, site, progression, and disease duration as well as with adiponectin serum level. We found that the *TG* genotype was significantly present in AA patients increasing its risk by about 5 folds, and the *G* allele significantly increased the risk by about 4 folds. Moreover, *ADIPOQ (rs2241766)TG* genotype carriers demonstrated significantly low adiponectin serum levels and a severe form of AA. Therefore, we suggested that *ADIPOQ* (rs2241766) gene polymorphism contributes not only to the development of AA, but also to its severity, that is mediated through dysregulation of adiponectin transcription.

Supporting these results, Saleh et al.^41^ revealed that *ADIPOQ* (rs2241766) mutant alleles were linked to diminished circulatory adiponectin levels in cases having myocardial infarctions. Furthermore, Al-Shaheri et al.^22^ found that *ADIPOQ*rs2241766 and rs3774261 SNPs increase the risk of atopic dermatitis. Previously, Wassel et al.^42^ reported that adiponectin SNPs rs17300539, rs182052, rs822393, rs9882205, and rs3774261 were strongly associated with adiponectin serum concentrations in whites.

The study limitations were: a) The small sample size, b) The study is structurally a case-control one, and c) The authors evaluated only a single adipokine rather than multiple ones.

## Conclusion

It seems that *ADIPOQ* (rs2241766) gene polymorphism (*TG* genotype and *G* allele) may modulate AA risk and contribute to the development of AA in Egyptian populations. Decreased circulating adiponectin levels may have a dynamic role in AA etiopathogenesis that could possibly be mediated via its anti-inflammatory effects. Adiponectin serum concentration can be considered a severity marker of hair loss in AA.

## Financial support

None declared.

## Authors’ contributions

Azza Gaber Antar Farag: Critical literature review; study conception and planning.

Eman Abd-Elfatah Badr labeeb: Data analysis and interpretation.

Banan Mohamed Gamal Abd-Elaty: Data collection

Nada Farag Elnaidany: Statistical analysis.

Mai Medhat Mohamed Ghanem: Data analysis and interpretation.

## Conflicts of interest

None declared.
